# Precuneus hyperexcitability mediates inflammatory-driven pain hypersensitivity following sleep disruption: a multimodal neuroimaging study

**DOI:** 10.3389/fimmu.2026.1744480

**Published:** 2026-05-04

**Authors:** Chao Li, Yang Wang, Kechong Zhou, Peng Zhang, Gang Chen, Xufeng Jiang, Yuhong Guo

**Affiliations:** 1 Department of Orthopedics, Xiangyang Central Hospital, Affiliated Hospital of Hubei University of Arts and Science, Xiangyang, Hubei, China; 2 Department of Urology, Xiangyang Central Hospital, Affiliated Hospital of Hubei University of Arts and Science, Xiangyang, Hubei, China

**Keywords:** functional MRI, general liner model, inflammation, pain hypersensitivity, sleep disruption

## Abstract

**Background:**

Sleep disruption(SD) has been shown to amplify inflammatory signaling and promote pain hypersensitivity, but how inflammation interacts with brain functional alterations to drive pain sensitization remains unclear. This study therefore aims to investigate inflammatory associated pain hypersensitivity induced by SD and characterize neural correlates for mediating neuroimmune-pain interactions.

**Materials and methods:**

This study employed two complementary designs: 1) A experimental design comparing forced awakening (FA) and uninterrupted sleep (US) groups, with polysomnography (PSG), task-fMRI, structural MRI, inflammatory cytokines, and quantitative sensory testing post-intervention; 2) A longitudinal cohort of chronic pain patients assessed pre- and 3-months postoperatively using neuroimaging, clinical pain metrics, and sleep quality indices (PSQI). Mass univariate analyses were performed to reveal differences in pain-elicited brain response between FA and US group. Structural and functional metrics were analyses within resultant brain regions using patient cohort. Multivariate correlations and mediation models were performed to test neuroimmune interactions.

**Results:**

FA subjects exhibited reduced total/slow-wave sleep, elevated IL-6, and lowered pain-thresholds versus US. Task-fMRI revealed hyperactivation in the precuneus and middle temporal gyrus during pain processing after FA. Serum IL-6 inversely correlated with pain thresholds and positively with precuneus activation. Mediation analysis demonstrated a mediating effect of precuneus hyperexcitability on the IL-6-pain hypersensitivity relationship. Longitudinal data from patient cohort complemented these findings, showing aberrant precuneus normalization correlated with improvement in pain and sleep quality.

**Conclusion:**

Across experimental and clinical cohorts, we identified the precuneus as a cortical hub linking sleep disruption, inflammation, and pain hypersensitivity, thereby revealing a novel neuroimmune pathway for sleep-related pain amplification.

## Introduction

The bidirectional relationship between sleep disturbances and pain perception constitutes a critical nexus in understanding chronic pain pathophysiology (1). Growing epidemiological evidence reveals that chronic pain patients experience comorbid sleep disorders, while sleep disruption independently predicts incident pain conditions with an increased risk (2). This intricate interplay is particularly evident in chronic pain conditions like rotator cuff injury (RCI), where disrupted scapulohumeral rhythm during lateral decubitus positioning creates a self-perpetuating cycle of nocturnal pain exacerbation and sleep discontinuity (3–5). While clinical observations consistently demonstrate that sleep disruption (SD) lowers pain thresholds and amplifies inflammatory signaling, the neural mechanisms through which these processes interact to drive central sensitization remain poorly characterized.

Recent advances have established systemic inflammation as a key mediator in SD-associated hyperalgesia. Emerging evidence suggests a bidirectional relationship between sleep disruption and systemic inflammatory responses (6, 7). Preclinical investigations employing animal models have mechanistically linked interleukin upregulation to enhanced nociceptive signaling through Toll-like receptor pathways (8, 9). This neuroimmune crosstalk provides a biological substrate for inflammation-potentiated pain processing. Furthermore, emerging neurophysiological evidence positions interleukin (IL) -6 as a pivotal signaling molecule in sleep disruption-mediated hyperalgesia. Experimental SD protocols induce elevations in circulating IL-6 that inversely correlate with pain thresholds in human participants (10), establishing a quantifiable neuroimmune relationship.

However, previous study has not disentangled inflammatory effects from direct neural consequences of sleep loss and lack of translational validation across experimental and clinical populations. This knowledge gap hinders the development of mechanism-targeted interventions for sleep-related pain exacerbation. The current study addresses these gaps through a dual-cohort design combining experimental sleep manipulation with longitudinal clinical assessment. We hypothesize that SD-induced functional alterations within brain associated with inflammatory-driven pain hypersensitivity. By integrating task-based fMRI, resting-state connectivity, and inflammatory biomarker profiling across controlled experimental and clinical longitudinal designs, this investigation elucidates the neural correlates of SD-related pain hypersensitivity. These findings would advance our understanding of how sleep disruption transitions acute inflammatory responses into sustained pain states through central nervous system sensitization, providing insights addressing sleep-related pain exacerbation.

## Materials and methods

### Participants

This investigation received institutional review board authorization from Local ethics committee (IRB ID: 2025-XY-019-15). Written informed consent was obtained from all subjects preceding experimental protocols. Forty-four healthy volunteers meeting eligibility requirements underwent task functional neuroimaging assessments: Absence of prior documented chronic nociceptive conditions, dyssomnia, or Axis I psychiatric disorders. Exclusionary parameters comprised: (1) Concurrent pain syndromes (including but not limited to widespread myofascial pain or inflammatory arthropathies); (2) DSM-V classified psychiatric comorbidities; (3) Central nervous system pathology (e.g., seizure disorders, cerebrovascular events); (4) Recent psychoactive substance misuse (≤12 months); (5) MRI contraindications (ferromagnetic implants, severe claustrophobia); (6) Pharmacological agents influencing monoaminergic transmission or sleep architecture within 28 days; (7) Decompensated systemic illness (HbA1c >9%, eGFR <30 mL/min/1.73m²). Participants were randomly allocated into uninterrupted sleep (US) and forced awakening (FA) groups using a pseudo-randomized sequence. Four participants withdrew from the experiment due to adverse effects associated with sleep disruption.

The investigation prospectively recruited 34 individuals with radiologically confirmed rotator cuff injuries (RCI) alongside 30 demographically matched asymptomatic controls. Post-enrollment quality control measures led to exclusion of three RCI participants: one due to incomplete longitudinal data and two demonstrating excessive head motion during neuroimaging acquisitions. RCI eligibility criteria include: (i) Adult surgical candidates (18–65 years) (ii) Unilateral supraspinatus pathology verified through combined clinical/imaging evaluation (MRI and musculoskeletal ultrasonography) (iii) Planned arthroscopic reconstruction within 6-week preoperative window (iv) Right-handed (v) Compliance with serial neuroimaging protocols (baseline & 3-month postoperative assessments). Patients were excluded with comorbid psychiatric disorders, long-term sedative/hypnotic or steroid use. Control subjects underwent identical vetting procedures as the task functional imaging cohort, maintaining methodological parity through application of parallel inclusion criteria. Final analytical sample comprised 31 RCI cases and 30 matched controls following attrition adjustments. All enrolled RCI patients received fully standardized perioperative care delivered by a single senior surgical team. This standardized clinical workflow included identical arthroscopic rotator cuff repair (ARCR) surgical procedures, a uniform postoperative multimodal analgesia protocol, and consistent standardized postoperative rehabilitation guidance across the entire cohort.

### Study pipeline

**Figure 1:**
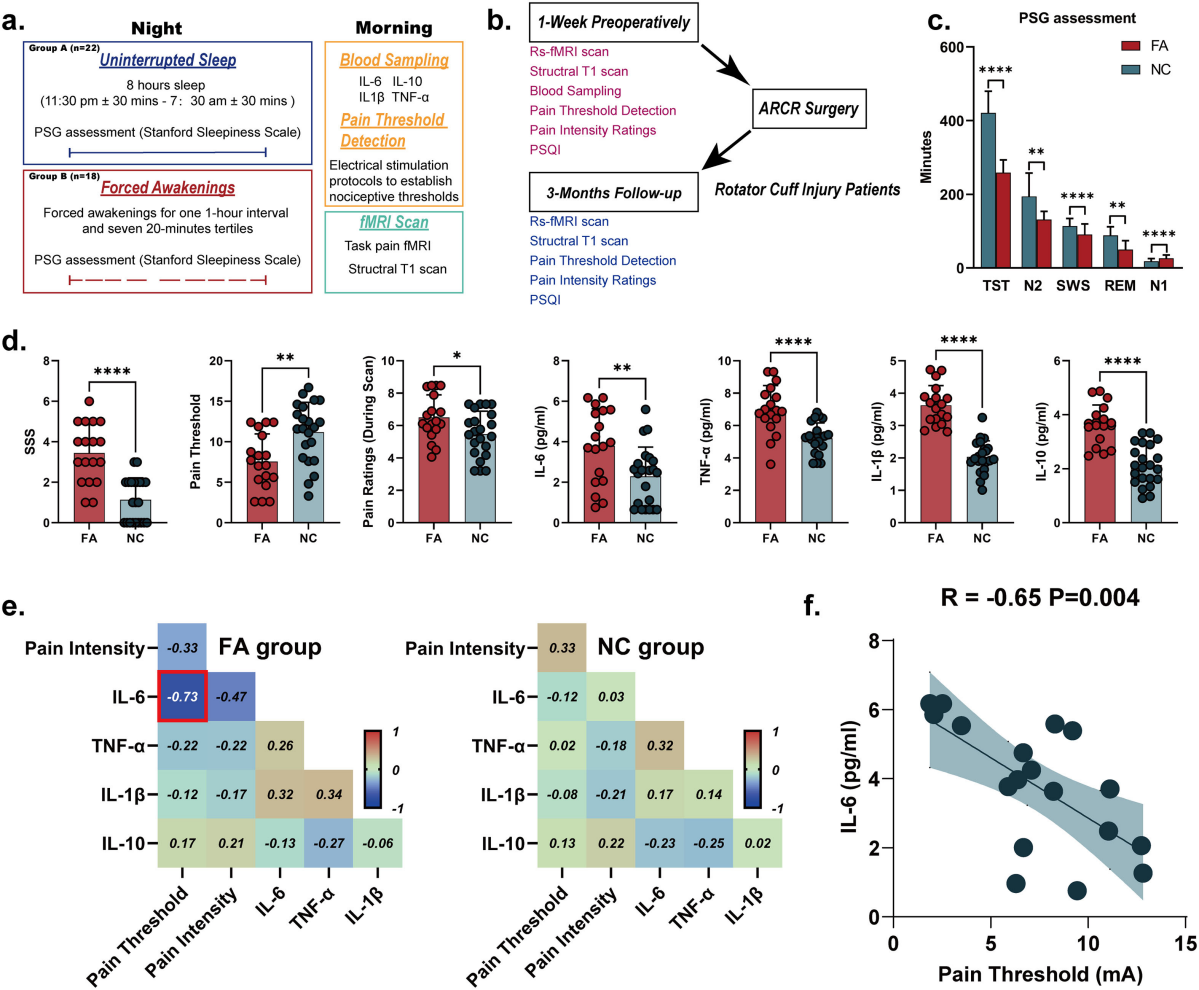
Experimental design and clinical-neuroimaging correlations. **(A)** Task-based fMRI protocol in sleep intervention cohorts. Participants were randomized into forced awakening (FA, n=18) and uninterrupted sleep (US, n=22) groups. Polysomnography (PSG) recordings were obtained during nocturnal sleep. Post-intervention assessments included: (1) task-fMRI with painful stimuli, (2) resting-state fMRI and T1-weighted structural MRI, and (3) serum inflammatory markers (IL-6, TNF-α) and quantitative sensory testing (pain threshold) measured prior to neuroimaging. **(B)** Longitudinal resting-state fMRI design in rotator cuff injury (RCI) cohort. Preoperative assessments comprised structural/functional MRI, visual analog scale (VAS) pain intensity, pain threshold, serum cytokines, and Pittsburgh sleep quality index (PSQI). Following arthroscopic rotator cuff repair (ARCR), postoperative evaluations at 3-month follow-up repeated neuroimaging and clinical metrics. **(C)** PSG-derived sleep architecture comparisons between FA and US groups, including total sleep time (TST), slow-wave sleep (SWS), N1, N2 and REM sleep duration. **(D)** Between-group differences in post-intervention outcomes: inflammatory markers (IL-6, TNF-α, etc.), psychophysical thresholds (pain threshold), and daytime sleepiness (Stanford sleepiness scale, SSS). **(E)** Multivariate correlation matrices of clinical measures. Heatmap intensity reflects Pearson correlation coefficients. **(F)** FA-specific correlation analysis demonstrating inverse relationship between serum IL-6 concentrations and pain thresholds after adjusting anxiety score measured by STAI scores. ARCR, arthroscopic rotator cuff repair; FA, forced awakening; IL, interleukin; PSQI, Pittsburgh sleep quality index; REM, rapid eye movement; SWS, slow-wave sleep; TNF, tumor necrosis factor; TST, total sleep time; US, uninterrupted sleep; VAS, visual analog scale.

For the task-fMRI cohort, a stratified block randomization design (block size = 4, **Supplementary Figure 1A**) was applied to allocate eligible healthy participants into the Forced Awakening (FA, final n=18) and Uninterrupted Sleep (US, final n=22) groups, with stratification by age, sex, BMI, and baseline sleep quality to ensure *a priori* balance in core baseline characteristics between groups. Independent samples t-tests confirmed no statistically significant differences in all baseline demographic, psychological, sleep, and pain-related characteristics between the final FA and US groups (all p > 0.05, detailed in **Table 1**). For the US condition, participants were given the opportunity for 8 hours of sleep without experimental disruption. For FA group, the present study used a previously described sleep disruption protocol. We chose sleep disruption (i.e., FA) based on previous work suggesting that this type of paradigm results in greater impairment of endogenous pain modulation compared to sleep restriction (11). Briefly, the night was divided into eight 1-hr intervals, with one randomly chosen interval in which no sleep was allowed. The remaining seven 1-hr intervals were further divided into 20-min tertiles, so that one tertile per hour interval was randomly selected for a forced awakening. Staffs were responsible for waking up participants at these randomly chosen times and ensuring that participants remained awake throughout the interval. The maximum sleep allowed for the FA night was 280 min (**Figure 1A**). Immediately following the controlled sleep disruption protocol, serum inflammatory cytokines (IL-6, IL-10, TNF-α, etc.) and pain detection thresholds were quantified at morning awakening. Subsequently, task-based fMRI data were acquired using a pressure-pain paradigm, structural T1-weighted imaging was also performed to control for neuroanatomical confounders.

**Table 1:** Demographic data of healthy participants for sleep disruption experiment.

Variables	FA (n=18)	US (n=22)	P value	Effect size
Age	34.1±7.1	35.8±12.4	0.61	Cohen’s d=0.16 (95%CI: -0.52, 0.84)
Sex	9/9	11/11	1	Cramer’s V=0.00
Years of Education	11.2±7.2	12.7±9.6	0.58	Cohen’s d=0.18 (95%CI: -0.50, 0.86)
Smoking history (Yes/No, n, %)	2(11.1)/16(88.9)	3(13.6)/19(86.4)	0.82	Cramer’s V=0.07
Alcohol consumption history (Yes/No, n, %)	3(16.7)/15(83.3)	4(18.2)/18(81.8)	0.90	Cramer’s V=0.04
Family history of chronic pain (Yes/No, n, %)	1(5.6)/17(94.4)	2(9.1)/20(90.9)	0.70	Cramer’s V=0.09
STAI-S	48.7±12.4	47.6±16.2	0.81	Cohen’s d=0.08 (95%CI: -0.56, 0.72)
STAI-T	49.2±13.6	47.1±11.9	0.61	Cohen’s d=0.17 (95%CI: -0.47, 0.81)
SSS	71.2±1.1	1.1±0.9	0.75	Cohen’s d=0.10 (95%CI: -0.54, 0.74)

FA, force awakenings; US, uninterrupted sleep.

The present study recruited patients with rotator cuff injury (RCI) as the clinical cohort complementary to the experimental sleep manipulation (**Supplementary Figure 1B**). RCI patients exhibit a well-characterized, high prevalence of sleep-pain comorbidity, which stems from the direct biomechanical interaction between RCI pathophysiology and lateral decubitus sleep posture (12). Chronic shoulder pain and impaired scapulohumeral rhythm, particularly during shoulder abduction that occurs in side-sleeping positions, induce sustained glenohumeral compression and nocturnal pain exacerbation (3, 13). This creates a robust, clinically relevant bidirectional cascade: pain directly disrupts sleep continuity and architecture, while chronic fragmented sleep in turn amplifies pain. This well-defined clinical phenotype establishes RCI as an optimal translational model to investigate whether the sleep disruption-inflammation-pain hypersensitivity pathway identified in our controlled experimental cohort is dysregulated in chronic sleep-pain comorbidity, and whether it predicts longitudinal clinical outcomes.

As delineated in **Figure 1B**, baseline measurements were systematically collected within 1 week preoperatively, including: Serum inflammatory cytokines, Neuroimaging profiles, Pain phenotyping and Sleep quality. At the 3-month postoperative follow-up after arthroscopic rotator cuff repair (ARCR), all participants underwent repeat assessments of Neuroimaging, Pain phenotyping and Sleep quality assessment.

### Behavior tests and blood sampling

Pain thresholds were detected using an electrical stimulation protocols similar to previous study (14). Bipolar cutaneous stimulation (0.2ms monophasic pulses; DS-7A stimulator, Digitimer Ltd) targeted the right deltoid region through a distal anode configuration (20mm inter-electrode distance), optimized for spatial summation while circumventing receptor adaptation. Sensory-discriminative thresholds were determined through a validated psychophysical paradigm employing sequential ascending stimulus series (4-trial blocks), with initial acclimatization trials discarded per protocol. Sensory detection threshold (SDT) represented the minimal perceptible current intensity, while pain detection threshold (PDT) was operationalized as the minimum current eliciting sharp, myelinated Aδ-fiber-mediated nociceptive perception, eliminating subjective pain magnitude estimation requirements. Preoperative pain chronicity was longitudinally mapped via Visual Analog Scale (VAS) recordings across quarterly intervals. Nocturnal dysfunction was objectively stratified using Pittsburgh Sleep Quality Index (PSQI) metrics, with scores >7 indicating clinically relevant sleep pathology. All behavioral assessments maintained ≥48-hour washout period preceding neuroimaging acquisitions to mitigate acute cognitive confounders. Post-surgical outcome tracking incorporated protocolized 90-day evaluations following arthroscopic repair. IL-6, TNF-α, IL-1β and IL-10 were selected as core mediators based on preclinical/clinical evidence for their key roles in sleep disruption-inflammation-pain crosstalk, covering pro-inflammatory and anti-inflammatory balance. Serum inflammatory mediators (TNF-α, IL-1β, IL-6, IL-10) were quantified via commercial chemiluminescent immunoassays (Aimplex^®^, Quantobio) following venous blood collection processed under cryogenic conditions: whole blood centrifuged at 3,000×g (4 °C) for 15 minutes with subsequent aliquots stored at -80 °C until batch analysis.

### Pressure pain task-fMRI paradigm

This investigation employed a block design via E-Prime 3.0 (Psychology Software Tools, Pittsburgh, PA). The pressure pain fMRI paradigm followed established protocols from previous study (15). The pressure pain task include two fMRI runs; each run contains 8 pseudorandomized trials with standardized 3 kg/cm² stimulus intensity. Each trial sequence initiated with a variable-duration rest period (10-15s) to minimize anticipation effects, followed by three distinct phases: 1) a 200ms auditory cue signaling impending stimulation, 2) a 5-10s pain anticipation interval, and 3) a 10s pressure pain application phase. Following stimulus offset, participants provided 10-15s dual pain assessments via MRI-compatible trackball-operated Visual Analogue Scales (0-100mm), evaluating both sensory intensity (“not painful” to “most imaginable pain”) and affective unpleasantness (“not unpleasant” to “most unpleasant experience”). The scale’s numerical values were intentionally obscured to prevent cognitive anchoring effects, with participants required to finalize responses within a 10-12s response window. This computerized assessment protocol ensured precise temporal synchronization with neuroimaging acquisition.

### Functional MRI data acquisition and preprocessing

Brain MRI data was obtained in 3T Discovery MR750w scanner (GE Healthcare) with a 32-channel phased-array head coil. Gradient-recalled echo planar imaging (GRE-EPI) sequence was employed to obtained functional MRI data. The scan parameters were: 44 axial slices (3 mm isotropic voxels), matrix=96×96, TR = 2000 ms/TE=30 ms, FOV = 240×240 mm². Task fMRI for pressure pain paradigm comprised 260 volumes over 8m40s, while resting-state fMRI utilized identical parameters with 300 volumes across 10 minutes. Brain T1 image were obtained using a magnetization-prepared rapid gradient echo (MPRAGE) sequence with following parameter for spatial normalization and cortical segmentation: TI = 900 ms, TR = 1900 ms/TE=2.52 ms, 176 sagittal slices with 1 mm³ isotropic resolution (FOV = 256×256 mm), flip angle=9°.

The task fMRI images were preprocessed using SPM12. The data was first 1) slice timing corrected, 2) Motion correction with acquisition of framewise displacement parameters, 3) Multimodal co-registration of functional data to individual T1 anatomy, and 4) Spatial normalization to MNI152 space via DARTEL registration. Finally, preprocessed data was spatial smoothed with 6mm FWHM isotropic Gaussian kernel to enhance signal-to-noise ratio. Resting-state fMRI preprocessing was conducted using DPARSFA following established protocols for neuropathic pain studies. The pipeline incorporated: 1) slice timing correction, 2) motion realignment with framewise displacement calculation, 3) co-registration to high-resolution T1 anatomy, and 4) spatial normalization to MNI152 space using DARTEL registration. Spatial smoothing (6mm FWHM Gaussian kernel) was applied post-normalization, except for regional homogeneity (ReHo) analysis where smoothing followed ReHo computation to preserve local connectivity patterns. Tissue segmentation (gray/white matter/CSF) was performed using unified segmentation, with subsequent nuisance regression of physiological signals (white matter/CSF mean signals) and motion parameters (24-parameter model). Temporal band-pass filtering (0.01-0.1 Hz) addressed low-frequency drift and high-frequency noise. The Artifact Detection Toolbox (ART v2.3) identified motion-contaminated volumes using dual criteria: framewise displacement >0.5mm or global signal Z-score >3.

### General liner model analyses

General linear model was conducted using SPM12 to characterize individual pain-elicited brain response. The designed matrix included: 1) painful stimuli (10s duration), 2) anticipating periods (5-10s), 3) post-stimulus periods (10-12s), and 4) rating periods. Six rigid-body motion parameters and their temporal derivatives were included as nuisance regressors, along with spike regressors for motion-contaminated volumes (FD >0.5mm). At the group level, one-sample t-tests with a family-wise error (FWE) correction at p<0.05 (voxel-level) were performed to obtained the group-level activation maps. This approach addressed multiple comparisons while maintaining sensitivity to detect pain-related activation patterns in the brain.

### Resting-state functional measurement and voxel-based morphometry

Regional spontaneous neural activity was quantified using two complementary metrics: Amplitude of Low-Frequency Fluctuations (ALFF) and Regional Homogeneity (ReHo). Analyses were conducted in DPARSFA following established protocols for pain neuroimaging studies. For ALFF computation: 1) Time series underwent detrending and band-pass filtering (0.01-0.1 Hz), 2) Fast Fourier Transform converted signals to frequency domain, 3) Square-root transformed power spectral density was averaged across 0.01-0.1 Hz, 4) Individual ALFF maps were standardized by global mean normalization to minimize inter-subject variability. ReHo analysis evaluated local BOLD synchronization using Kendall’s Concordance Coefficient (KCC) within 27-voxel cubes (central voxel + 26 neighbors). KCC values were z-transformed relative to whole-brain mean and smoothed (6mm FWHM) to meet Gaussian field assumptions. Notably, spatial smoothing was applied post-normalization to preserve local connectivity patterns.

Dynamic regional metrics (dALFF/dReHo) were computed using DPABI-integrated Temporal Dynamic Analysis modules. For temporal feature extraction, our protocol implemented a sliding window approach which is a validated technique for capturing temporal fluctuations and mapping cerebral metric variability. In resting-state dynamic assessments, window duration selection represents a critical determinant requiring balance between temporal resolution (for detecting rapid neurodynamic shifts) and measurement reliability (for robust regional activity characterization). Previous neuroimaging investigations have employed variable window spans (10–180 seconds). Our analytical framework adopted a 30-TR window configuration (60-second duration). To quantify ALFF/ReHo temporal fluctuations, we derived voxel-wise standard deviation (SD) values across all temporal windows for each participant. Global mean normalization was subsequently performed to mitigate intersubject variability. Final preprocessing involved spatial smoothing using a 6-mm FWHM Gaussian kernel to enhance signal-to-noise ratio while preserving neuroanatomical specificity.

Brain structural evaluations employing T1-weighted sequences implemented voxel-based morphometry (VBM) protocols to quantify cerebral gray matter volume. Our processing pipeline incorporated CAT12/SPM12 architectures for high-resolution image optimization, featuring: 1) Manual AC-PC alignment following quality assurance screening to optimize spatial normalization; 2) Tissue segmentation (CSF/WM/GM) after intensity normalization to address magnetic field inhomogeneities; 3) Advanced spatial normalization via DARTEL-based nonlinear warping with Jacobian modulation for intensity correction; 4) Total intracranial volume (TIV) quantification through compartmental summation of segmented tissue probabilities. The morphometrically refined brain images underwent voxel-wise z-score transformation relative to cohort means, generating normalized parametric maps for subsequent multimodal analyses. These preprocessing procedures ensured neuroanatomical comparability while controlling for individual cranial capacity variations through TIV-adjusted intensity scaling.

### AHBA processing

Transcriptomic data integration from the Allen Human Brain Atlas (AHBA) implemented a standardized processing framework through the abagen toolbox (https://github.com/netneurolab/abagen), adapting established neuro-genomic protocols (16). Microarray-derived transcriptional profiles from the AHBA repository which containing postmortem adult brain specimens with preprocessed expression matrices, underwent hemisphere-specific curation, retaining 1028 left-hemispheric cortical samples based on tissue availability. Probe-gene mapping leveraged current NCBI Entrez annotations to ensure genomic annotation fidelity. Following quality assurance protocols, we excluded probes exhibiting subthreshold detection rates (>50% samples with intensity below background noise levels). To enhance biological validity, RNA-seq concordance filtering prioritized probes demonstrating maximal cross-platform correlation for each gene. Normalization employed robust sigmoid transformation to mitigate platform-specific technical variability across specimens. This multistage curation yielded standardized transcriptional signatures for 10,185 protein-coding genes per tissue sample, establishing a molecular atlas for multimodal integration with neurodynamic parameters. The refined gene-brain mapping matrix subsequently informed spatial correlation analyses with functional neuroimaging biomarkers, facilitating mechanistic interpretations of neurophysiological patterns through molecular signatures.

### Statistical analyses

#### Behavior signatures and sleep-pain interactions in FA and US

To investigate the aberrant behavior profile in FA compared to US group, two sample t tests were performed to reveal the differences in polysomnography (PSG) assessments, pain threshold, pain ratings during scan and serum level of inflammatory cytokines (IL-6, IL-10, IL-1β, TNF-α). After regressing out the variance explained by the STAI State Anxiety Inventory (SAI) score for all behavioral metrics, pairwise Pearson correlation analyses were performed separately in the FA and US groups. Mass-univariate analyses were performed to reveal the brain activation differences between FA and US group within a grey matter mask, with age, sex, years of education, S-AI score as covariates, and the statistical maps were corrected using family‐wise‐error (FWE) method at cluster level (p<0.05). Subsequently, the average β value within the resultant brain cluster was extracted from each subject and correlated with PT and inflammatory cytokines concentration (e.g., IL-6, TNF-α). To test the hypothesized associative pathway between significantly correlated brain functional alterations and pain-related behavioral signatures, mediation analyses were performed separately for the forced awakening (FA) and uninterrupted sleep (US) groups with S-AI scores as covariates. To mitigate statistical bias from the limited sample size, the Freedman-Lane Independent Error Resampling permutation test with 20,000 iterations was performed to validate the mediation effects (17). A statistically significant mediation effect was determined with a concurrent permutation p-value < 0.05. It should be noted that this analysis evaluates a theoretically derived associative mediational pathway, rather than confirming definitive causal relationships.

#### Clinical profile and brain functional/structural abnormalities in RCI vs. HC

The baseline measurement in terms of pain threshold, PSQI, pain intensity, IL-6, IL-10, IL-1β, TNF-α for RCI and HC patients were compared. Longitudinal change in PSQI was also compared to reveal the recovery trajectory following ARCR surgery in RCI patients. Subsequently, RCI patients were allocated into PSQI high and PSQI low group based on their preoperative PSQI score using a cut-off value of 7 (18). Non-parametric Mann-Whitney U tests on paired preoperative-to-postoperative change scores were performed to compare the recovery trajectories of pain threshold and pain intensity following ARCR surgery between two groups. Based on the significant differences in pain-evoked activation cluster identified from the FA vs. US group comparison and the central role of the precuneus in pain modulation, the bilateral precuneus, defined by the Anatomical Automatic Labeling (AAL) template, was selected as the region of interest (ROI) for subsequent brain functional and structural analyses. The static ALFF/ReHo, dynamic ALFF/ReHo and grey matter volume were compared within these ROIs. The means of these functional and structural measures were extracted and correlated with clinical variables preoperatively. For longitudinal data, pair-wise t tests were performed to reveal the static ALFF/ReHo, dynamic ALFF/ReHo and grey matter volume differences between pre and postoperative fMRI data in RCI patients. To control for the confounding effects of anxiety and depression, we first regressed out the variance explained by the Hospital Anxiety and Depression Scale (HADS) depression (HADS-D) and anxiety (HADS-A) subscale scores from the pre-to-postoperative change values of each neuroimaging metric. The standardized residuals of these change metrics were then extracted and used for Pearson correlation analyses with the pre-to-postoperative change values of clinical variables in RCI patients.

#### Transcriptional correlates for sleep-pain interactions in FA

Transcriptional neuroimaging associations with nociceptive processing patterns were examined through Spearman’s ρ correlation analyses between transcriptional profiles and task-based neural contrast maps (NA vs US groups). To address multiplicity in genome-wide univariate screening (n=10,185 genes), we implemented a dual-threshold framework: 1) Spatial autocorrelation-adjusted permutation testing (10,000 Monte Carlo iterations generating empirical null distributions) with cluster-defining threshold of p<0.05; 2) Family-wise error control via Bonferroni adjustment (α=4.91×10^-6^). Significant loci underwent multi-scale functional annotation through ToppGene Suite (https://toppgene.cchmc.org), identifying enriched biological processes via hypergeometric testing with Benjamini-Hochberg FDR correction (q<0.05). Cellular specificity of pain-sleep interference-associated transcripts was probed using WebCSEA (https://bioinfo.uth.edu/webcsea/submit.php). This multimodal analytic cascade ensured rigorous control of both spatial and genomic confounds while bridging molecular pathways to neurofunctional phenotypes.

## Results

### Behavior profile following sleep disruption

Forced awakenings (FA) cohort exhibited alterations across both sleep quality and pain modulation domain. Polysomnography confirmed that FA participants (vs. uninterrupted sleep, US) had reduced total sleep time (TST), decreased N2-stage sleep, slow-wave sleep (SWS), and rapid eye movement (REM), alongside elevated N1-stage (P < 0.001, **Figure 1C**). This aberrant architecture was also shown with higher level of daytime somnolence (e.g., Stanford Sleepiness Scale, **Figure 1D**). Critically, FA participants demonstrated pain hypersensitivity, with significantly lower pain thresholds and amplified pain intensity during pressure pain stimuli (**Figure 1D**). Concurrently, FA individuals showed elevated pro-inflammatory cytokines, including IL-6, TNF-α, and compensatory IL-10, suggesting a systemic inflammatory surge post-sleep disruption (**Figure 1D**). Strikingly, inflammatory-pain coupling diverged between groups (**Figure 1E**). In FA participants, IL-6 levels were inversely correlated with pain thresholds (R = -0.73, P < 0.001; **Figure 1E**) and remained robust following adjustment for SAI scores (R = -0.65, P = 0.004, **Figure 1F**), implicating IL-6 as a correlates of pain sensitization. Conversely, US participants showed no significant cytokine-pain associations, underscoring sleep continuity’s protective role against neuroimmune-driven hyperalgesia (Demographic data could be found in **Table 1**).

### Neural correlates for FA induced hyperalgesia

**Figure 2:**
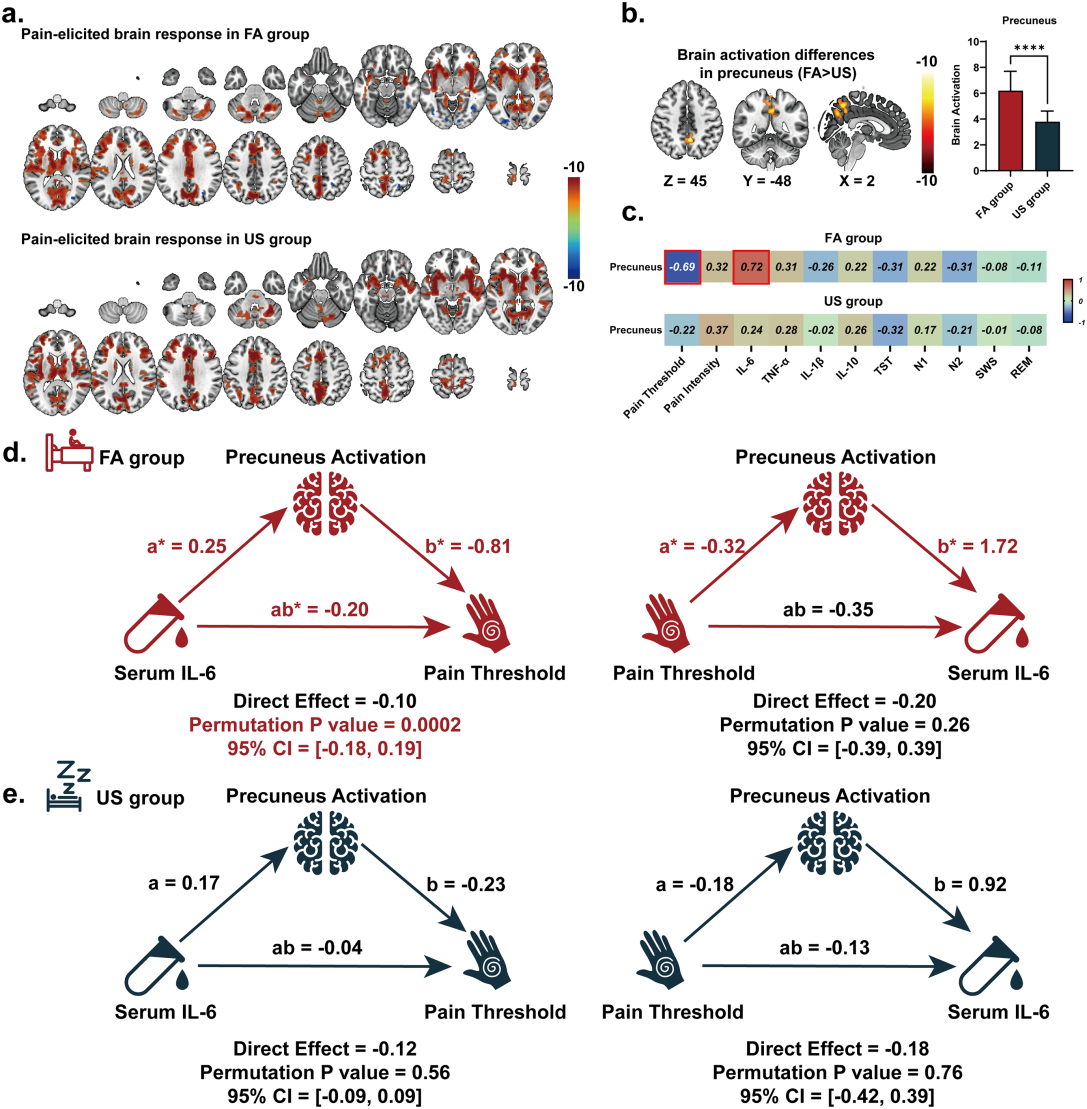
Sleep disruption-modulated pain neuro-signatures and mediation pathways. **(A)** Task-evoked fMRI activation maps in response to painful stimuli. Thresholded at cluster-level family-wise error (FWE)-corrected P<0.05. Upper: forced awakening (FA) group (n=18); lower: uninterrupted sleep (US) group (n=22). Color bars indicate T-scores. **(B)** Between-group differences in precuneus cortex and middle temporal gyrus. **(C)** Multivariate brain-clinical correlations. Heatmaps display Pearson’s r values between regional activation (precuneus/MTG) and pain, sleep, inflammatory related measures **(D)** mediation model testing IL-6→precuneus activation→pain threshold pathway and alternative pain threshold→precuneus activation→IL-6 pathway. REM, rapid eye movement; SWS, slow-wave sleep; TNF, tumor necrosis factor; TST, total sleep time; US, uninterrupted sleep.

Both groups exhibited significant activations in the primary somatosensory cortex (S1), insular cortex, thalamus, Rolandic operculum, precuneus, and cerebellum during nociceptive stimulation (**Figure 2A**). Between-group contrasts revealed that FA patients demonstrating significantly enhanced activation (family-wise error corrected p<0.05) in the left precuneus (**Figure 2B**, **Table 2**). Notably, differential brain-behavior relationships emerged between cohorts (**Figure 2C**). In FA participants, precuneus hyperactivity showed strong negative correlation with pressure pain thresholds (Pearson’s R = -0.69, P <0.001) and positive association with serum IL-6 concentrations (R = 0.72, P<0.001). Conversely, no significant brain-behavior correlations were observed in US group (all P > 0.1), consistent with normative pain processing and inflammatory profiles. To examine potential pathways linking inflammatory markers with precuneus hyperactivity and pain hypersensitivity in FA, we conducted mediation analyses using permutation tests (20,000 iterations). The primary model testing IL-6 to precuneus activation to pain threshold demonstrated significant partial mediation (ab = -0.20, 95% CI [-0.18, 0.19], P = 0.0002) (**Figure 2D**). Contrary to alternative directional models, reverse mediation testing (IL-6 as independent variable, pain threshold as outcome) revealed no significant mediating role of precuneus activation (**Figure 2D**.). Crucially, these relationships were absent in US group, revealing a significant tripartite association among serum IL-6 concentrations, precuneus hyperactivation, and hyperalgesia.

**Table 2:** Brain regions exhibited significant group differences pain elicited brain activation between force awakenings and uninterrupted sleep group.

Brain region	Number of voxels	F value	MNI coordinates (x,y,z)		
Precuneus	244	6.3	2	-48	45

### Clinical signature in chronic pain patients with sleep disturbance

**Figure 3:**
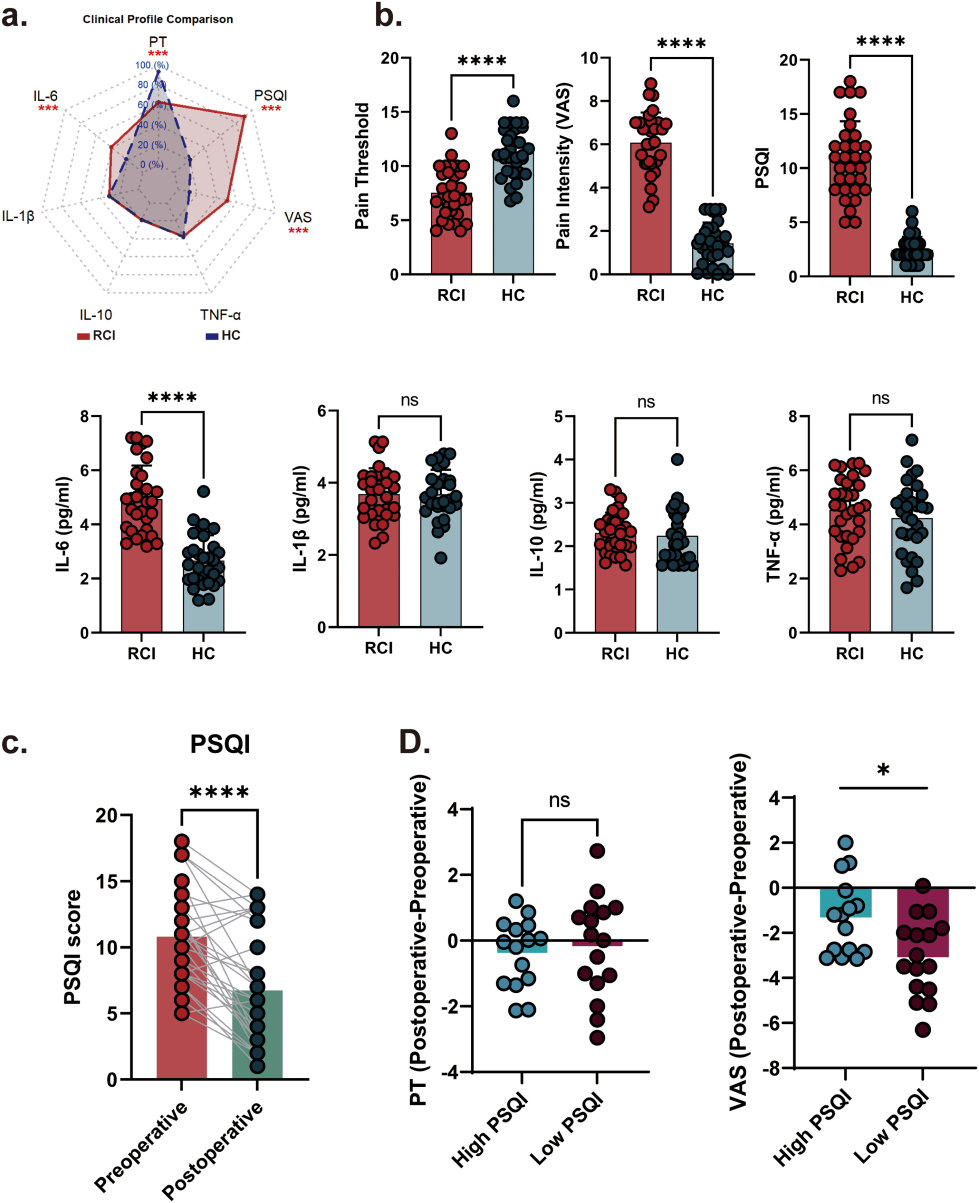
Multidimensional clinical signatures and sleep-pain interactions in rotator cuff injury (RCI). **(A)** Radar plot of clinical domain patterns. **(B)** Baseline cross-sectional comparisons. RCI patients demonstrated significant elevation in serum IL-6, alongside reduced pain thresholds and impaired PSQI scores. **(C)** Longitudinal sleep quality trajectories. Post-arthroscopic repair (ARCR), PSQI scores showed progressive improvement from preoperative to 3-month follow-up. **(D)** Between group differences in changes of pain threshold and VAS score following surgery. ARCR, arthroscopic rotator cuff repair; HC, healthy controls; IL, interleukin; PSQI, Pittsburgh sleep quality index; TNF, tumor necrosis factor; VAS, visual analog scale.

Building on our findings of the acute sleep disruption model, we next sought to validate this axis in clinical scenarios of chronic sleep disturbance. To this end, we collected clinical data from patients with rotator cuff injury (RCI), a cohort selected based on robust epidemiological evidence demonstrating a high prevalence of sleep disturbances in this population. Demographic characteristics of the study cohort are presented in **Table 3**. Distinct clinical profiles emerged between RCI patients and HCs in our cohort (**Figure 3A**). Quantitative analyses revealed significantly reduced pain thresholds, elevated Pittsburgh Sleep Quality Index (PSQI) scores, and increased IL-6 levels in RCI patients compared to HCs (**Figure 3B**). These findings collectively suggest concomitant dysregulation of nociceptive processing, sleep architecture, and systemic inflammation. Notably, no intergroup differences were observed in IL-10, IL-1β, or TNF-α levels (**Figure 3B**), implying a dissociation between chronic inflammatory states, as evidenced by IL-6 elevation, and acute-phase proinflammatory cytokine activity. Following surgery, the sleep quality was improved in FA group (**Figure 3C**). Furthermore, we calculated the changes in mechanical pain threshold (PT) and pain intensity between the preoperative and postoperative periods and performed between-group comparisons of these change scores. The high-PSQI group exhibited a significant reduction in pain intensity (**Figure 3D**).

**Table 3:** Demographic data of patient cohort in current study.

Variables	RCI (n=31)	HC (n=30)	P value	Effect size
Age	53.1±8.5	53.2±11.1	0.96	Cohen’s d=-0.01 (-0.62, 0.60)
Sex	17/17	15/15	1	Cramer’s V=0.00
Years of Education	12.1±8.3	11.7±7.6	0.84	Cohen’s d=0.05 (-0.57, 0.67)
Duration of Symptom (Months)	9.5±3.1	NA	NA	NA
Smoking history (Yes/No, n, %)	5(14.7)/29(85.3)	4(13.3)/26(86.7)	0.75	Cramer’s V=0.05
Alcohol consumption history (Yes/No, n, %)	6(17.6)/28(82.4)	5(16.7)/25(83.3)	0.80	Cramer’s V=0.03
Family history of chronic pain (Yes/No, n, %)	4(11.8)/30(88.2)	3(10.0)/27(90.0)	0.73	Cramer’s V=0.06
HADS-D	7.2±5.6	4.2±3.9	0.01	Cohen’s d=0.62 (95%CI: 0.13, 1.11)
HADS-A	7.6±6.2	4.6±3.2	0.02	Cohen’s d=0.60 (95%CI: 0.11, 1.09)

RCI, Rotator cuff injury; HC, Healthy control; NA, Not Applicable.

### Dysregulated central pain modulation and longitudinal recovery patterns in chronic pain patients

**Figure 4:**
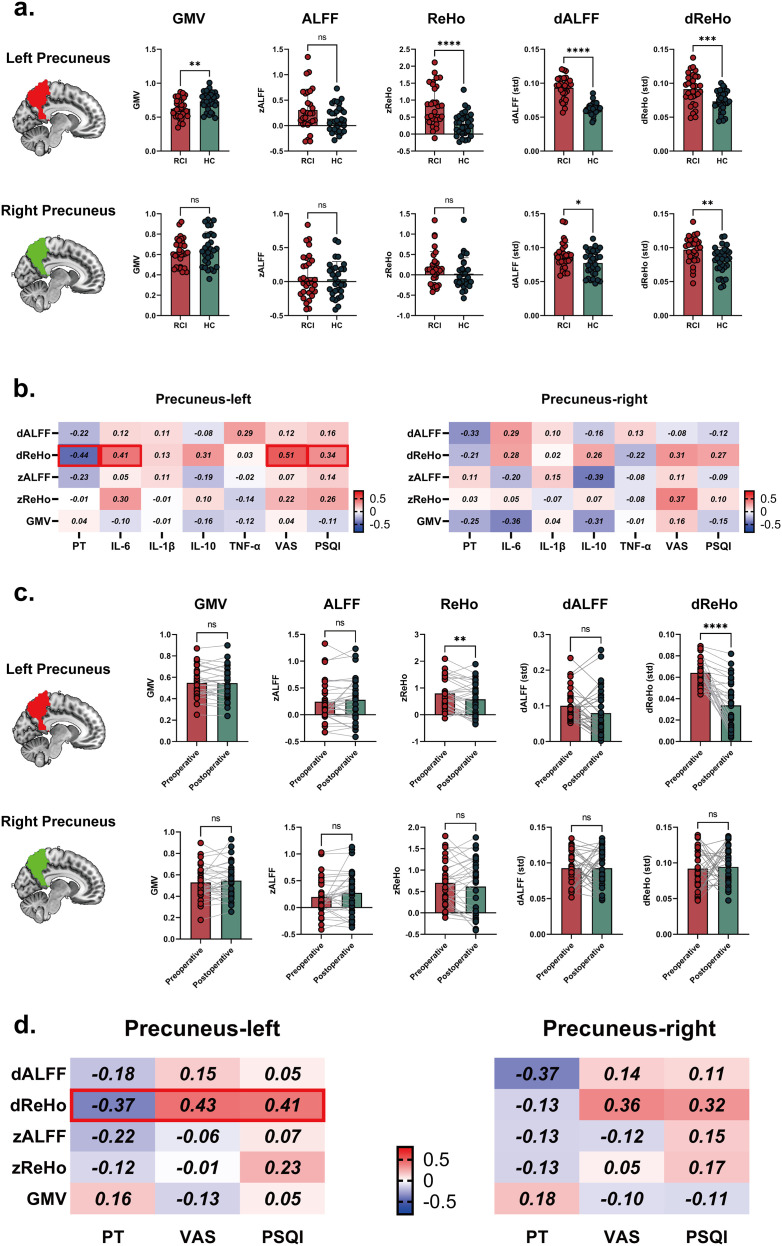
Resting-state fMRI biomarkers of central sensitization and longitudinal recovery patterns in rotator cuff injury. **(A)** Multidimensional neural oscillatory signatures. Differences in grey matter volume, static and dynamic amplitude of low-frequency fluctuation (sALFF) and regional homogeneity (sReHo) differences between RCI vs healthy controls. **(B)** Clinico-neural correlation patterns within precuneus in RCI group. **(C)** Postoperative rs-fMRI plasticity. Longitudinal changes within precuneus for grey matter volume, static and dynamic ReHo and ALFF measures. **(D)** Recovery trajectory predictors. Correlation coefficients between changes in brain measures and changes in clinical measures.

Having identified the precuneus as a key region mediating sleep-pain modulation, supported by prior literature documenting its critical role in pain modulation and association with pain sensitivity, we further performed region of interest (ROI) analyses of the bilateral precuneus to investigate functional and structural alterations between the RCI and healthy controls (HC) groups. Within these ROIs, multimodal analyses integrating static/dynamic amplitude of low-frequency fluctuations (ALFF), regional homogeneity (ReHo), and grey matter volume (GMV) revealed significant functional alterations in RCI patients compared to HC. In the left precuneus, RCI patients demonstrated significant GMV reduction alongside concurrent functional hyperactivity, characterized by elevated static ReHo, as well as increased dynamic ALFF and dynamic ReHo variability. In the right precuneus, RCI group exhibited preserved structural integrity and comparable static functional metrics. Notably, RCI patients showed increases in dynamic ALFF and dynamic ReHo variability. Correlation analyses revealed significant positive correlation between dynamic ReHo variability in left precuneus and IL-6 (R = 0.41), pain intensity (R = 0.51), PSQI (R = 0.34); and inversely correlated with pain threshold (R = -0.44), after adjusting the depression and anxiety level measured by HADS. While the right precuneus demonstrated no significant associations between grey matter volume (GMV), static/dynamic functional indices (ALFF/ReHo), and clinical variables. Postoperative longitudinal assessments revealed that, in the left precuneus, surgical intervention induced significant functional normalization, manifesting as reduced static and dynamic ReHo In the right precuneus, surgical intervention induced significant functional normalization of both dynamic ALFF and ReHo (**Figure 4C**). Intriguingly, precuneus dynamic ReHo variability reduction correlated with clinical improvement: positive associations with pain intensity decline and sleep quality enhancement, and negative correlation with pain threshold elevation (**Figure 4B**).

### Transcriptional correlates for molecular signatures of brain activation patterns induced by FA

**Figure 5:**
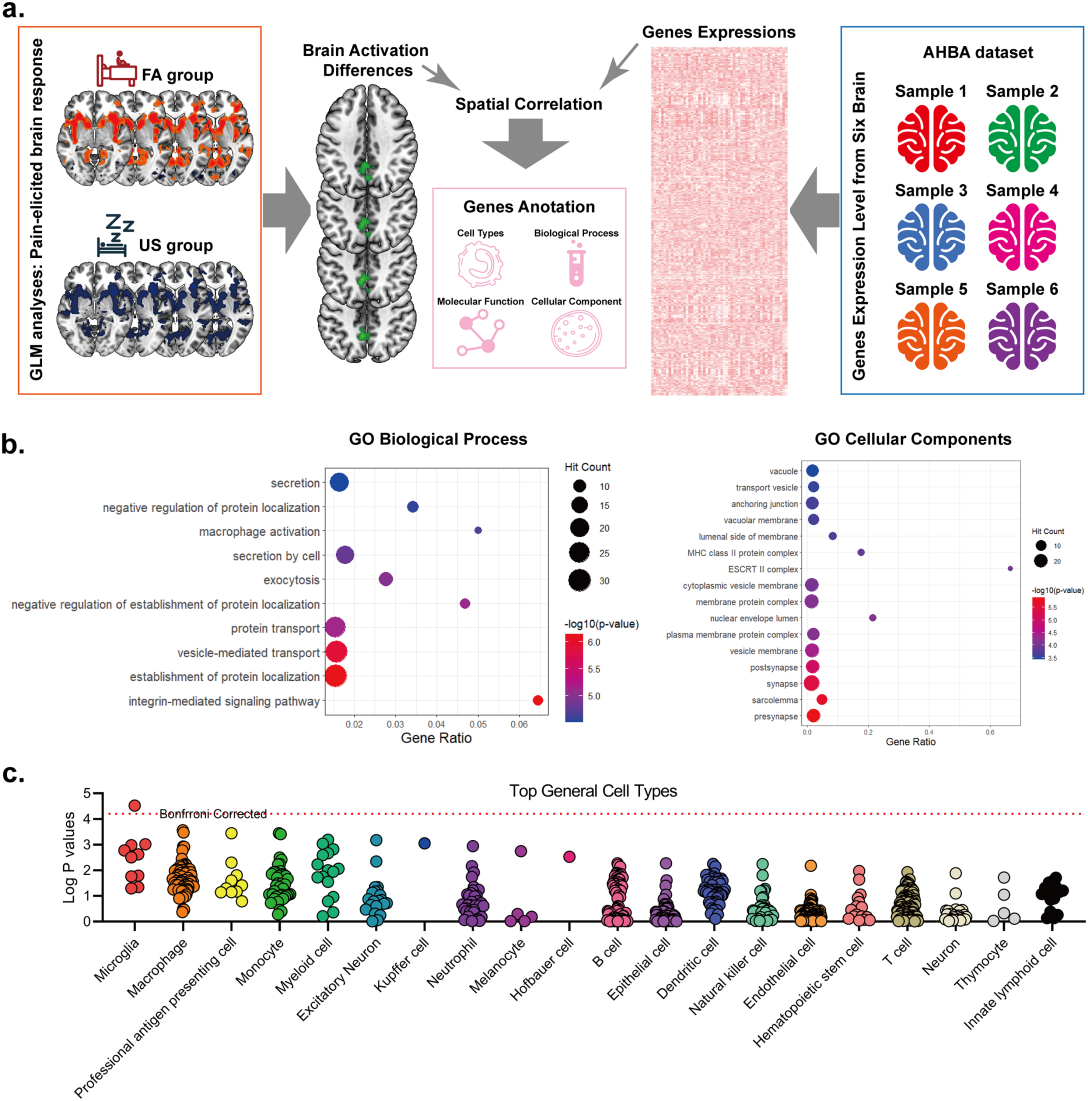
Transcriptome-neuroimaging integration reveals microglial-specific molecular signatures of sleep-pain interaction. **(A)** Multimodal integration pipeline. Neuroimaging-derived statistical maps of pain-evoked activation differences (FA vs US) were spatially correlated with gene expression data from the Allen human brain atlas (AHBA). Genes surviving Bonferroni correction (Padj<0.01) were selected for downstream functional annotation. **(B)** Functional enrichment of sleep-modulated genes. Gene ontology (GO) analysis identified significant enrichment in: Biological processes: integrin-mediated signaling pathway, Cellular components: presynaptic active zone. **(C)** Cell type-specific expression analysis indicates that the related genes are mainly expressed in microglia under a threshold controlled by Bonferroni correction.

**Figure 5A** illustrates the integration framework linking cortical transcriptional signatures to group-level neurofunctional divergences (FA vs US). Cortical-level analyses revealed 158 genes exhibiting spatial expression covariance with task activation contrasts. Biological process analyses identified overrepresentation of regulatory secretory mechanisms (exocytosis, vesicle-mediated transport) and immune-neural interactions (macrophage activation, protein localization modulation) (**Figure 5B**). Cellular compartment mapping demonstrated enrichment in intracellular trafficking apparatus (vacuolar/transport vesicle systems) and neuropil stabilization modules (anchoring junction complexes) (**Figure 5B**). Cell-type specificity interrogation via cross-tissue expression profiling showed microglial predominance among significant genes (Bonferroni-adjusted p<0.05, **Figure 5C**), suggesting neuroinflammatory mediation of observed neurofunctional disparities.

## Discussion

This multimodal neuroimaging investigation reveals a significant association between precuneus hyperexcitability and inflammatory-related pain hypersensitivity in the context of both acute and chronic sleep disruption. By integrating experimental sleep manipulation in healthy adults with longitudinal clinical assessments in chronic pain patients, our findings suggest that precuneus dysfunction as a neural correlates of disrupted sleep transitions inflammatory responses into sustained pain states.

Our experimental findings extend previous reports linking sleep disruption to elevated IL-6 levels and reduced pain thresholds by demonstrating that precuneus hyperactivity functionally couples inflammatory signaling to nociceptive processing. The negative association between circulating IL-6 levels and nociceptive thresholds corroborates animal evidence demonstrating sleep disruption-induced priming of monocyte reactivity and subsequent amplification of pro-inflammatory signaling (19, 20). Chronic sleep disruption has been established to produce sustained IL-6 elevations that correlate with self-reported daily pain intensity in clinical populations (21). Our investigation extends current knowledge by revealing that controlled sleep disruption activates upstream inflammatory pathways in humans, with inflammatory mechanisms serving as critical mediators of hyperalgesia responses. Notably, experimental induction of systemic inflammation through endotoxin produces multimodal hypersensitivity to pain stimuli across quantitative sensory testing paradigms (22). Epidemiological analyses further substantiate this biological link, demonstrating that elevated inflammatory markers predict reduced tolerance thresholds in standardized cold pressor testing (23, 24). Clinical investigations across heterogeneous chronic pain conditions, including fibromyalgia (25), knee osteoarthritis (26), complex regional pain syndrome (27), and postamputation neuropathy (28), have consistently demonstrated elevated circulating proinflammatory markers relative to pain-free controls. Beyond modulating nociceptive processing, amplified inflammatory signaling contributes to the pathogenesis of chronic inflammatory diseases and modifies infectious disease trajectories, reflecting IL-6’s as a molecular regulator that orchestrates both adaptive immune activation and innate inflammatory pathways (29).

Notably, the nociceptive signal transmission and modulation underlying sleep disruption-induced hyperalgesia are not confined to higher-order brain regions alone, as the spinal cord, a key lower-order central nervous structure, exerts a pivotal role in the initiation and propagation of nociceptive signals. Accumulating preclinical evidence (30) has demonstrated that sleep disruption can robustly activate spinal microglia and astrocytes, the primary immune cells in the spinal cord parenchyma (31); such activation promotes the local release of pro-inflammatory cytokines (e.g., IL-6, TNF-α) in the spinal dorsal horn and induces the sensitization of nociceptive neurons in this region. This spinal-level sensitization effectively lowers the activation threshold of nociceptive signaling pathways, thereby facilitating the conduction of peripheral nociceptive inputs to higher-order brain regions including the precuneus, and ultimately contributing to the development of central sensitization and pain hypersensitivity (32). It is important to note that the current study focused exclusively on higher-order brain regions due to technical constraints that preclude the simultaneous acquisition of high-quality neuroimaging data from the spinal cord and the brain within the same experimental paradigm, which limits our ability to characterize the integrated functional interactions between spinal cord and cortical regions in sleep-pain-neuroinflammation crosstalk. In current study, the novel mechanistic insight lies in identifying the precuneus, a key hub of the default mode network (DMN) (33, 34), as the neural interface translating peripheral inflammatory signals into central sensitization (35, 36). This finding resonates with emerging evidence of precuneus involvement in migraine chronification and fibromyalgia pathophysiology (37, 38), where its functional connectivity alterations correlate with both spontaneous pain and hyperalgesia. The DMN comprises interconnected cortical regions implicated in introspective cognition and intrinsic homeostasis monitoring. Notably, its functional architecture demonstrates dynamic antagonism with executive control network (ECN) engagement during goal-directed tasks, where enhanced DMN suppression correlates with superior neurocognitive efficiency (39, 40). In the present study, the precuneus, rather than classic pain network regions including the insula, anterior cingulate cortex (ACC), and thalamus, emerges as the key hub of the sleep-inflammation-pain axis. The sensory-discriminative pain network, which consists of the insula, ACC and thalamus and primarily mediates the transmission and initial processing of nociceptive signals (41), stands in contrast to the precuneus, which subserves the integrative functions of the self-referential/default-mode network. Notably, the precuneus is not a primary nociceptive region but a higher-order integrator that coordinates interoceptive input, affective salience, and immune signaling; this unique functional property enables it to transduce peripheral inflammatory signals into central sensitization, a critical step that is absent in the direct nociceptive processing of classic pain-related brain regions (42, 43).

Our exploratory mediation analyses revealed associative patterns consistent with a hypothesized sequential pathway linking sleep disruption to reduced nociceptive thresholds via IL-6 elevation and subsequent precuneus hyperactivation. This putative associative hierarchy offers a testable framework for future interventions targeting sleep-related pain exacerbation. Notably, these mediation analyses were exploratory and limited by a small sample size; the marginal reverse model result may reflect insufficient statistical power to detect weak effects, rather than confirming no reverse association. Future validation in larger cohorts with additional inflammatory markers and multi-omics data is needed to clarify these potential associations and underlying neuroimmune regulatory networks.

Integration of our neuroimaging data with transcriptomic data from the Allen Human Brain Atlas (AHBA) identified a significant spatial correlation between FA-evoked aberrant neural activation patterns in the precuneus and the cortical expression profiles of microglia-enriched genes. This transcriptomic finding provides biological plausibility for the involvement of the precuneus in the sleep-inflammation-pain pathway, aligning with prior work implicating microglia in neuroimmune interactions (44), and in particular their proposed role in modulating nociceptive signaling via cytokine release and synaptic remodeling in chronic pain conditions (45). Importantly, these AHBA-based analyses are strictly hypothesis-generating and do not provide direct mechanistic proof of the observed associations and require further functional validation.

**Figure 6:**
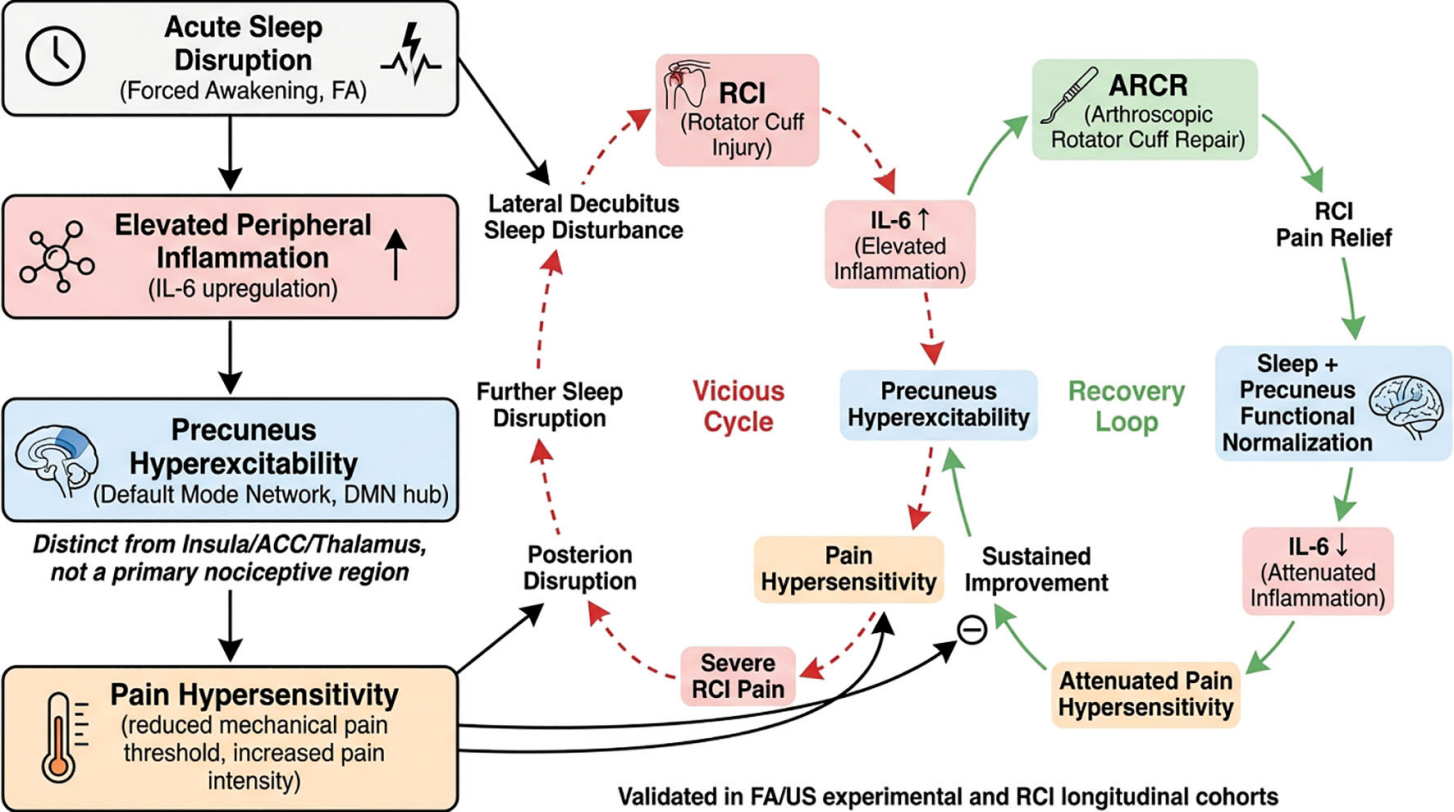
The schematic model of current study.

The functional specialization of the precuneus in SD-induced hyperalgesia may stem from its unique neuroanatomical position as a cortical nexus integrating interoceptive, affective, and self-referential processing (46). Neuroimaging studies in primary headache disorders have consistently implicated precuneus dysregulation in pain catastrophizing and altered body perception, cognitive-affective dimensions that our pressure pain paradigm likely engaged through its threat anticipation components (47). The observed hyperactivation during both pain anticipation and stimulation phases suggests that sleep disruption amplifies precuneus involvement across multiple stages of nociceptive processing. Our resting-state data further revealed increased precuneus activity, potentially reflecting enhanced DMN activity that merit detailed examination in future analyses. The clinical relevance of these findings is underscored by longitudinal data showing that preoperative sleep disruption predicts postoperative pain trajectories in RCI patients, a population where sleep-pain interactions are particularly salient due to biomechanical constraints during lateral decubitus positioning. From a therapeutic perspective, our results position the precuneus as a promising neuromodulation target for conditions where sleep disruption and inflammation synergistically drive pain persistence. Sleep disruption induces precuneus hyperactivation across nociceptive processing stages (with elevated resting-state activity possibly reflecting enhanced DMN activity), and preoperative sleep disruption predicts postoperative pain trajectories in RCI patients, underscoring the precuneus as a promising neuromodulation target for pain persistence driven by synergistic sleep disruption and inflammation. As a core hub of the SD-inflammation-pain axis, the precuneus has key translational value for mechanism-targeted pain management (**Figure 6**), with three clinical intervention strategies: (1) Precuneus/DMN-targeted non-invasive neuromodulation (TMS/tDCS) to normalize hyperexcitability and disrupt the IL-6-precuneus-pain cycle; (2) Sleep-focused interventions (e.g., CBT-I) to mitigate SD, reduce systemic inflammation (e.g., IL-6) and suppress precuneus hyperactivation, with combined surgical/physical therapy improving outcomes in RCI and musculoskeletal pain patients; (3) Precuneus neuroimaging and peripheral inflammatory biomarkers (e.g., serum IL-6) to identify high-risk patients and guide targeted interventions for personalized pain management.

Several limitations warrant consideration. First, our experimental sleep disruption model (forced awakenings) differs mechanistically from clinical insomnia, potentially limiting direct translation to chronic pain populations. While the FA protocol effectively induces sleep disruption, real-world sleep disturbances often involve complex interactions between physiological and psychological factors not captured in laboratory settings. Additionally, while no significant pre-intervention differences in clinical, psychological, or pain-related outcomes were observed between groups, the absence of baseline fMRI scans precludes definitive confirmation that the detected group differences in brain activation resulted solely from the intervention. Future studies with pre-post intervention fMRI designs may help clarify this issue. Second, the focus on IL-6 as a primary inflammatory mediator overlooks potential contributions from other non-cytokine inflammatory markers. Third, the 3-month postoperative follow-up in our clinical cohort captures early but not long-term pain outcomes, necessitating extended observation periods to assess whether precuneus changes predict pain chronification. Also, we did not collect other pain related behavior measurements (e.g., from the Pain Catastrophizing Scale) in this cohort, future study with a more comprehensive assessment is needed. Our Future muti-center studies are needed with a larger sample size for validating the generalizability of our results. Fourth, this study lacks animal experiments to validate the causal relationship between sleep disruption-induced inflammation, precuneus activity and pain hypersensitivity, animal models enable precise experimental manipulation but are unavailable to us due to facility limitations. Future collaborative animal studies will measure inflammatory markers, precuneus activity and pain-related behaviors under sleep disruption and verify mechanisms via targeted intervention. Furthermore, our sample size for mediation analyses is small for making a causal proof, future studies with larger sample size is needed for validate our findings. Last, as noted, AHBA transcriptomic integration is hypothesis-generating, with a small donor pool and spatial autocorrelation potential. Future work should validate these findings via independent human cohort transcriptomic data and *in vivo* animal studies.

## Conclusion

In conclusion, this study establishes precuneus hyperexcitability as a critical neural mechanism through which sleep disruption amplifies inflammatory signaling to drive pain hypersensitivity. By bridging experimental and clinical approaches, we provide evidence that this DMN hub serves as a cortical integrator of neuroimmune-nociceptive interactions. These findings advance our pathophysiological understanding of sleep-pain comorbidity while identifying novel targets for mechanistic interventions in chronic pain disorders.

## Data Availability

The raw data supporting the conclusions of this article will be made available by the authors, without undue reservation.
