# Supplementary material for: Precuneus hyperexcitability mediates inflammatory-driven pain hypersensitivity following sleep disruption: a multimodal neuroimaging study
Source: Front Immunol. 2026 May 4;17:1744480. doi: 10.3389/fimmu.2026.1744480 (PMC13180543; doi:10.3389/fimmu.2026.1744480)
Supplement: Supplementary file 1 [file DataSheet1.docx]

**Supplementary methods**

**Functional fMRI data acquisition and preprocessing pipeline**

Functional MRI data were acquired on a 3T Discovery MR750w scanner (GE Healthcare) using a 32-channel head coil. Whole-brain coverage was achieved with gradient-recalled echo planar imaging (GRE-EPI) sequences using the following parameters: 44 axial slices (interleaved acquisition; 3 mm isotropic voxels; matrix=96×96; TR=2000 ms/TE=30 ms; flip angle= 54°; FOV=240×240 mm²; anterior-posterior phase encoding). For the pressure pain paradigm, 260 volumes were acquired over 8m40s (initial 10 dummy scans discarded for signal stabilization), while resting-state fMRI used identical parameters (300 volumes/10 minutes; 10 dummy scans discarded).

High-resolution T1-weighted images were obtained with magnetization-prepared rapid gradient echo (MPRAGE) sequence (176 sagittal slices; 1 mm³ isotropic; FOV=256×256 mm; TI=900 ms; TR=1900 ms/TE=2.52 ms; flip angle=9°). All preprocessing was implemented in MATLAB R2021b (MathWorks) using SPM12. Task fMRI processing included: 1) slice-timing correction; 2) motion realignment with framewise displacement (FD) calculation (participants with FD >2.0mm excluded, n=0); 3) multimodal co-registration to individual T1 anatomy; 4) DARTEL normalization to MNI152 space; and 5) spatial smoothing (6mm FWHM Gaussian kernel). The Artifact Detection Toolbox (ART v2.3) identified motion-contaminated volumes (FD >0.5mm or global signal Z>3) across all functional scans. Resting-state data were processed similarly using DPARSFA, with nuisance regression of physiological signals (24-parameter motion, white matter/CSF) and temporal band-pass filtering (0.01–0.1 Hz).

**Supplementary Figures**


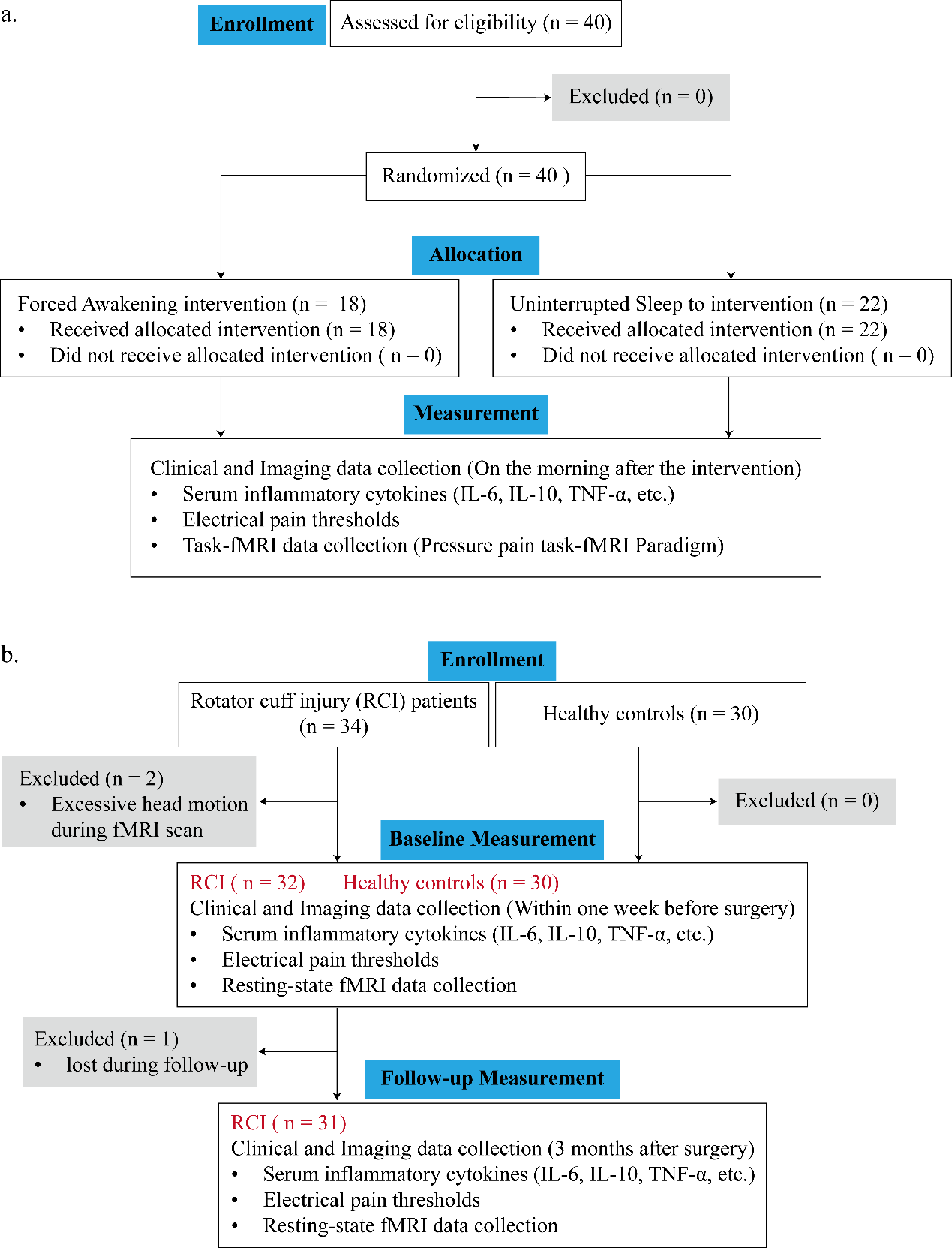


**Sup-Figure 1.** CONSORT flow diagrams. a: Participant flow for the task-fMRI substudy. b: Participant flow for the resting-state fMRI substudy.
